# Isolation, Pathogenicity, and Genomic Analysis of a Virulent *Aeromonas salmonicida* ST2 Strain from *Thymallus arcticus grubei*

**DOI:** 10.3390/biology15191791

**Published:** 2026-10-08

**Authors:** Ran Zhao, Di Wang, Jing Wang, Jianqiang Zhu, Yongquan Zhang, Kai Ma, Shaowu Li

**Affiliations:** Key Laboratory of Aquatic Animal Diseases and Immune Technology of Heilongjiang Province, Heilongjiang River Fisheries Research Institute, Chinese Academy of Fishery Sciences, Harbin 150070, China; zhaoran@hrfri.ac.cn (R.Z.);

**Keywords:** *Aeromonas salmonicida*, pathogenicity, T3SS, cold-water fish, *Thymallus arcticus grubei*

## Abstract

*Aeromonas salmonicida*, the causative agent of furunculosis, poses a serious threat to salmonid aquaculture, but its pathogenicity in rare cold-water fish species remains insufficiently characterized. This study reports the first isolation and characterization of a pathogenic *A. salmonicida* strain (Ag001) from diseased *Thymallus arcticus grubei* in China. Multilocus sequence typing (MLST), together with 16S rRNA, *gyrB*, and *vapA* gene analyses and phylogenetic characterization, identified the isolate as *A. salmonicida* subsp. *salmonicida* ST2. In intraperitoneal challenge assays using six bacterial dose groups and PBS controls, Ag001 showed high virulence toward *T. arcticus grubei* [LD50: 3.08 × 10^3^ CFU/mL (95% CI: 9.42 × 10^2^–1.01 × 10^4^ CFU/mL), equivalent to 7.16 × 10^1^ CFU/g (95% CI: 2.19 × 10^1^–2.34 × 10^2^ CFU/g)] and *Oncorhynchus mykiss* [LD50: 3.16 × 10^4^ CFU/mL (95% CI: 1.19 × 10^4^–8.38 × 10^4^ CFU/mL), equivalent to 3.46 × 10^1^ CFU/g (95% CI: 1.31 × 10^1^–9.17 × 10^1^ CFU/g)]. Comparative histopathological examination of spleen, liver, and kidney tissues from diseased and healthy fish revealed splenic necrosis, hepatic degeneration, and renal tubular damage in diseased fish. Genomic analysis identified complete T2SS and T3SS secretion systems, along with eight virulence-associated genes including *aerA*, *hlyA*, and *vapA*, suggesting that secretion systems and virulence factors may contribute to pathogenicity. Antimicrobial susceptibility testing showed susceptibility to enrofloxacin, neomycin, and doxycycline, but resistance to several other tested agents. These findings provide information on the host range, pathogenicity, genomic virulence features, and potential treatment options for *A. salmonicida* ST2 in cold-water aquaculture systems.

## Simple Summary

*Aeromonas salmonicida* is a bacterial pathogen that can cause severe disease and mortality in salmonid fish. However, its effects on Arctic grayling (*Thymallus arcticus grubei*) in China remain poorly understood. We isolated strain Ag001 from diseased Arctic grayling and examined its identity, pathogenicity, genome, and antimicrobial susceptibility. Multilocus sequence typing classified Ag001 as sequence type 2, linking it to a lineage associated with disease outbreaks in salmonid fish. Infection experiments showed that Ag001 was highly pathogenic to both Arctic grayling and rainbow trout (*Oncorhynchus mykiss*), causing severe damage to the spleen, liver, and kidney. Genome analysis identified two major protein-delivery systems involved in bacterial infection. The chromosome encoded a type II secretion system, whereas a plasmid carried a type III secretion system. Together with several toxin- and virulence-related genes, these systems may contribute to the high pathogenicity of Ag001. The strain was susceptible to enrofloxacin, neomycin, and doxycycline under laboratory conditions. This study provides the first report and comprehensive characterization of *A. salmonicida* from diseased Arctic grayling in China, expanding its known host range and providing useful information for disease surveillance and control in cold-water aquaculture.

## 1. Introduction

*Aeromonas salmonicida*, a Gram-negative bacterium and the causative agent of furunculosis, poses a major threat to the global aquaculture industry [1,2]. The pathogen is mainly transmitted horizontally through contact with infected or carrier fish and exposure to contaminated water. The pathogen primarily infects salmonid fish species, including *Salmo salar* and *Oncorhynchus mykiss* [3], frequently causing high mortality and substantial economic losses. In recent decades, its host range has expanded considerably, with infections also reported in marine and freshwater non-salmonid species including *Gadus morhua* [4], *Scophthalmus maximus* [5], *Sparus aurata* [6], *Solea senegalensis* [7], and *Petromyzon marinus* [8]. The pathogen typically enters the host through damaged skin, gills, or intestinal mucosa, leading to systemic infection characterized by hemorrhagic septicemia, tissue necrosis, and furuncle formation [3,8,9]. Clinical manifestations vary among host species, with salmonids presenting typical furuncles [10], whereas non-salmonid hosts more frequently present ulcerative skin lesions [3,11]. *A. salmonicida* is well adapted to cold-water environments and is therefore regarded as an important bacterial pathogen in cold-water aquaculture systems [2,12].

Arctic grayling (*Thymallus arcticus grubei*), a cold-water salmonid endemic to the Heilongjiang River basin in northeastern China, has recently attracted increasing interest as a potential aquaculture species because of its high commercial and nutritional value [13]. However, long-term overfishing and habitat degradation have resulted in substantial population declines and increasing conservation concern for this species [14]. Consequently, efforts to develop sustainable aquaculture practices have intensified to support both commercial production and population conservation. *T. arcticus grubei* exhibits optimal growth within a relatively narrow temperature range of 7–15 °C, which overlaps with the favorable growth conditions of *A. salmonicida*, potentially increasing the risk of bacterial disease outbreaks in cultured populations.

Recently, a disease outbreak characterized by skin ulceration and hemorrhagic symptoms occurred in cultured *T. arcticus grubei* in Heilongjiang Province, China. In this study, we isolated the dominant bacterial pathogen, designated Ag001, from diseased fish and identified it as *A. salmonicida*. Artificial infection experiments confirmed its pathogenicity, representing the first reported case of *A. salmonicida* infection in *T. arcticus grubei* in China. We further characterized the isolate using integrated phenotypic, molecular, and genomic analyses to investigate its genetic features and pathogenic potential. The findings of this study expand current knowledge of bacterial pathogens affecting cold-water fish species and provide a scientific basis for disease surveillance and health management in both aquaculture and conservation programs for *T. arcticus grubei*.

## 2. Materials and Methods

### 2.1. Histopathological Analysis and Pathogen Isolation of Diseased T. arcticus grubei

Diseased *T. arcticus grubei* (10.5 ± 0.5 cm, mean ± SD) were sampled in a farm located in Heilongjiang Province, China. The diseased fish exhibited bleeding symptoms on the body surface, with bleeding spots at the base of the fins, scales, and head. Additional clinical signs included exophthalmia and distinct ulcerative furuncles.

For histopathological analysis, spleen, liver, and kidney tissues were collected from diseased *T. arcticus grubei* and healthy control fish (*n* = 3 fish per group). No tissue samples were pooled. The tissues were fixed in Bouin’s solution and dehydrated through a graded ethanol series prior to paraffin embedding. Sections were cut using a rotary microtome, stained with hematoxylin and eosin (H&E), and observed under a light microscope. For bacterial isolation, samples were aseptically collected from the kidney, spleen, and external lesions of five symptomatic diseased *T. arcticus grubei* using a sterile loop, and streaked onto Tryptone Soya Agar (TSA) plates for culture at 18 °C over 36 h. Predominant colonies were screened and subcultured on fresh TSA plates under identical incubation conditions. Single colonies were inoculated into Tryptic Soy Broth (TSB) and incubated at 18 °C for 24 h. Cultures were preserved at −80 °C in TSB supplemented with 20% (*v*/*v*) sterile glycerol. The predominant isolate from kidneys was labeled as Ag001.

### 2.2. Physiological, Biochemical, and Molecular Identification of Ag001

Prior to characterization, the purified Ag001 was revived from −80 °C onto TSA and incubated at 18 °C for 36 h. Gram staining was performed, and physiological and biochemical characteristics were determined using the BD Phoenix M50 system (BD, Sparks, MD, USA). Genomic DNA of Ag001 was extracted using a commercial bacterial genomic DNA extraction kit (TIANGEN Biotech, Beijing, China). This strain was molecularly identified by 16S rRNA and *gyrB* genes. The primers used were as follows: 27F (5′-AGAGTTTGATCCTGGCTCAG-3′)/1492R (5′-GGTTACCTTGTTACGACTT-3′) for 16S rRNA and gyrBF (5′-GAAGGCCAAGTCGGCCGCCAG-3′)/gyrBR (5′-ATCTTGGCATCGCCCGGGTTTTC-3′) for *gyrB* [15]. To further classify the subspecies of strain Ag001, A-layer typing was conducted based on sequence polymorphisms in the *vapA* gene [16]. Sequence similarity searches of the obtained sequences were performed using BLAST 2.17.0 via the NCBI database. Reference sequences were selected from closely related BLAST matches and representative *Aeromonas* species or subspecies available in GenBank, and their accession numbers are shown in the phylogenetic trees. Phylogenetic analysis was carried out using the neighbor-joining method implemented in MEGA 7.0. The reliability of the tree topology was evaluated by bootstrap analysis with 1000 replicates, and bootstrap values are shown as percentages at the nodes.

### 2.3. Multilocus Sequence Typing (MLST)

MLST was performed based on six housekeeping genes (*gyrB*, *groL*, *gltA*, *metG*, *ppsA*, and *recA*), following the protocol described in the PubMLST *Aeromonas* database. The primers used for the polymerase chain reaction (PCR) were obtained from the PubMLST *Aeromonas* spp. database [17]. PCR amplification of these genes was carried out, and the resulting sequences were visualized using DNAMAN for sequence assembly and editing. The verified sequences for each housekeeping gene of strain Ag001 were then analyzed using the PubMLST *Aeromonas* spp. database.

### 2.4. In Vivo Experimental Infection Study

Healthy *O. mykiss* (91.4 ± 6.5 g, mean ± SD) and *T. arcticus grubei* (4.3 ± 0.6 g, mean ± SD) were obtained from a fish farm in Ning’an City, Mudanjiang, Heilongjiang Province, China. Fish sex and exact age were not recorded in this study. The fish were acclimated for one week prior to the experiment. During acclimation, fish were maintained in 360 L tanks at approximately 150 fish per tank. Water temperature was maintained at 14 ± 0.5 °C, with dissolved oxygen at approximately 7.5–10.0 mg/L and pH at 7.0–7.5. A 12 h light:12 h dark photoperiod was maintained for all groups. Fish were fed a commercial formulated diet twice daily at a daily ration of approximately 2% of body weight. Approximately one-third of the tank water was replaced every two days.

Before the infection experiment, five additional fish of each species, *O. mykiss* and *T. arcticus grubei*, were randomly selected for bacteriological examination to confirm their pathogen-free status. These fish were not included in the subsequent infection experiment. For infection assays, fish were randomly allocated into seven groups (*n* = 10 fish per group). Each treatment group was maintained in a single tank containing 10 fish. Randomization was performed at the individual-fish level before fish were assigned to the corresponding treatment tanks. Fish of comparable body size were used, and all experimental groups were maintained under the same environmental and husbandry conditions to minimize potential confounding effects.

Six groups were intraperitoneally injected with serial concentrations of Ag001 (1 × 10^3^–1 × 10^8^ CFU/mL for *O. mykiss* and 1 × 10^2^–1 × 10^7^ CFU/mL for *T. arcticus grubei*), with an injection volume of 100 μL per fish. Fish injected with 100 μL of sterile PBS alone served as the control group. Mortality and clinical signs were recorded at the individual-fish level, and individual-fish mortality data were used for LD50 estimation. Samples were not pooled. Each dose group contained 10 fish. No formal a priori sample-size calculation was performed. Mortality and clinical signs were recorded daily for 7 days. Moribund fish used for bacterial re-isolation and surviving fish sampled at the end of the trial were euthanized with tricaine methanesulfonate (MS-222, 200 mg/L) before tissue collection. The LD_50_ and its 95% confidence interval were estimated using the improved Karber’s method [18]. LD_50_ values expressed in CFU/g were calculated as follows: LD_50_ (CFU/g) = LD_50_ (CFU/mL) × injection volume (0.1 mL)/mean body weight (g). The corresponding 95% confidence intervals expressed in CFU/g were converted using the same formula. To fulfill Koch’s postulates, kidney samples from moribund fish were subjected to re-isolation of the tested bacterium. No blinding or masking was applied during treatment administration, mortality and clinical-sign assessment, histopathological evaluation, or data analysis.

### 2.5. Analysis of Antimicrobial Susceptibility and Screening of Virulence-Associated Genes

Antimicrobial susceptibility of Ag001 was determined using the broth microdilution method according to CLSI guidelines [19]. Eight antibiotics were tested using commercial 96-well plates (Fosun Diagnostics, Shanghai, China). Bacterial suspensions were adjusted to a 0.5 McFarland standard and diluted to approximately 5 × 10^5^ CFU/mL in Mueller-Hinton (MH) broth. Aliquots (50 μL) were added to each well and incubated at 28 °C for 24 h. The MIC was determined as the lowest antimicrobial concentration that inhibited visible bacterial growth. MIC values were interpreted as sensitive, intermediate, or resistant based on the criteria listed in the CLSI MIC standard [20].

The presence of nine virulence-associated genes [aerolysin (*aerA*), zonula occludens toxin (*zot*), Dnases (*dna*), hemolysin (*hlyA*), cytotoxic enterotoxin (*act*), cytotonic enterotoxins (*alt*), S-layer protein (*sp*), lipase (*lip*), and A-layer (*vapA*)] was analyzed by PCR. The reaction parameters followed the corresponding references listed in Table 1.

### 2.6. Genome Sequencing and Analysis

Genomic DNA was extracted using the Wizard^®^ Genomic DNA Purification Kit (Promega, Madison, WI, USA). Whole-genome sequencing was conducted on the Illumina NovaSeq 6000 and PacBio Sequel IIe platforms (Majorbio, Shanghai, China). De novo assembly of the reads was performed with SOAPdenovo2 [27]. Plasmid replicons were identified using PLASMe v1.1 and further annotated by BLAST searches against the PLSDB database. Functional annotation against the EggNOG, NR, Swiss Prot, Pfam, Gene Ontology (GO), and Kyoto Encyclopedia of Genes and Genomes (KEGG) databases was carried out via the Majorbio Cloud Platform [28]. The genomic locations of secretion-system genes were determined according to their coordinates on the assembled chromosome and plasmid replicons, which allowed the T3SS gene cluster to be assigned to plasmid A.

## 3. Results

### 3.1. Clinical Examination and Histopathological Characterization of the Diseased T. arcticus grubei

Diseased *T. arcticus grubei* exhibited typical clinical signs, including ulcerative hemorrhagic lesions on the body surface, particularly along the lateral body region (Figure 1A). Hyperemia and hemorrhage were also observed at the base of the dorsal fin (Figure 1B). In addition, bilateral exophthalmia accompanied by subcutaneous cranial hemorrhage was observed (Figure 1C).

Histopathological analysis showed marked tissue alterations in multiple organs. In the spleen, splenocytes were loosely arranged, with focal necrosis, cytoplasmic vacuolization, and nuclear fragmentation (Figure 2B). The liver exhibited congestion, disorganization of hepatic cords, and inflammatory cell infiltration (Figure 2D). The diseased group had obvious renal vacuoles, disordered renal tubular structure, irregular luminal morphology, disordered arrangement of epithelial cells, partial cell degeneration and necrosis, unclear cell boundaries, and scattered microhemorrhagic foci (Figure 2F).

### 3.2. Physiological and Biochemical Characterization

The physiological and biochemical characteristics of Ag001 are revealed in Table 2. The isolate was positive for L-arabinose, glucose, D-fructose, and maltose, and negative for D-arabinose, adonitol, sorbitol, and L-rhamnose. These characteristics were consistent with the report of *A. salmonicida* in Bergey’s Manual of Systematic Bacteriology.

### 3.3. Sequence Analysis of the 16S rRNA, gyrB and vapA Gene

The 16S rRNA, *gyrB* and *vapA* sequences of Ag001 obtained by PCR amplification have been uploaded to NCBI with GenBank accession numbers PX664495, PX682281, and PZ122746, respectively. Sequence analysis showed that the 16S rRNA gene shared 99.9% identity with *A. salmonicida* (AB027544.2), while the gyrB gene exhibited 100% identity with *A. salmonicida* (KC244775.1). Phylogenetic analysis based on 16S rRNA and gyrB genes clustered Ag001 within the *A. salmonicida* clade (Figure 3 and Figure 4). The results of subspecies identification showed that Ag001 belongs to the subsp. *salmonicida*, as it is clustered with typical strains of *A. salmonicida* subsp. *salmonicida* BG and SS-Sk (Figure 5).

### 3.4. MLST Analysis of Ag001

Six housekeeping genes of Ag001 were successfully amplified and sequenced for MLST analysis. The allelic profile of Ag001 was identified as *gyrB*-2, *groL*-2, *recA*-2, *metG*-2, *gltA*-2, and *ppsA*-2. This profile corresponded to sequence type ST2 in the PubMLST database (Table 3).

### 3.5. Experimental Infections

No mortality was observed in the control groups throughout the experimental period. In *O. mykiss*, final mortality was 10/10, 10/10, 10/10, 7/10, 3/10, and 0/10 fish at concentrations of 1 × 10^8^, 1 × 10^7^, 1 × 10^6^, 1 × 10^5^, 1 × 10^4^, and 1 × 10^3^ CFU/mL, respectively (Figure 6). The LD_50_ was 3.16 × 10^4^ CFU/mL (95% CI: 1.19 × 10^4^–8.38 × 10^4^ CFU/mL), equivalent to 3.46 × 10^1^ CFU/g (95% CI: 1.31 × 10^1^–9.17 × 10^1^ CFU/g). In *T. arcticus grubei*, final mortality was 10/10, 10/10, 8/10, 8/10, 3/10, and 1/10 fish at concentrations of 1 × 10^7^, 1 × 10^6^, 1 × 10^5^, 1 × 10^4^, 1 × 10^3^, and 1 × 10^2^ CFU/mL, respectively (Figure 7). The LD_50_ was 3.08 × 10^3^ CFU/mL (95% CI: 9.42 × 10^2^–1.01 × 10^4^ CFU/mL), equivalent to 7.16 × 10^1^ CFU/g (95% CI: 2.19 × 10^1^–2.34 × 10^2^ CFU/g).

### 3.6. Genome Analysis of Strain Ag001

The genome of strain Ag001 consists of 4,885,720 bp with a GC content of 58.27%. The final assembly comprised four circular replicons, including one chromosome (4,648,003 bp) and three plasmids of 144,753 bp (plasmid A), 87,348 bp (plasmid B), and 5616 bp (plasmid C), respectively (GenBank accession No. JBVPZQ000000000.1). Read-mapping coverage was 100% for both the Illumina and PacBio datasets, and the PacBio sequencing depth was 83.42×. CheckM estimated genome completeness at 100% with 0.58% contamination, while BUSCO analysis showed 100% completeness. A total of 4518 protein-coding genes, 107 tRNA genes, and 28 rRNA genes were predicted (Figure 8A). Of the 4518 predicted protein-coding genes, 3720 (82.34%) were annotated in the COG database, with Metabolism representing the largest functional category (43.2%) (Figure 8B). A total of 3526 protein-coding genes were annotated in the KEGG database. Among these, the largest number was assigned to Metabolism (2321 genes), followed by Environmental Information Processing (474 genes) (Figure 8C).

### 3.7. Genomic Secretion Systems and Virulence Profiling

Genome analysis predicted the presence of genes associated with multiple secretion systems, including T1SS, T2SS, T3SS, T6SS, the Tat secretion system, and the Sec-SRP secretion system. Among them, the complete T2SS genes were located on the chromosome, whereas the complete T3SS gene cluster was identified on plasmid A. Furthermore, Virulence Factor Database (VFDB) screening confirmed the presence of key virulence-associated genes encoded in the genome, including *exeM*, *atsD*, *mshO*, *flpF*, *flgE*, *flrA*, *flpJ*, and *fimF* (Table 4). In addition, nine common virulence-associated genes of *Aeromonas* were examined by PCR. Ag001 was positive for eight genes (*aerA*, *act*, *zot*, *dna*, *sp*, *lip*, *vapA,* and *hlyA*), whereas *alt* was not detected (Figure 9 and Figure S1).

### 3.8. Antimicrobial Susceptibility

The antimicrobial susceptibility results are presented in Table 5. Strain Ag001 was susceptible to enrofloxacin, neomycin, and doxycycline, with MIC values of 0.5, 2, and 0.25 μg/mL, respectively. The isolate showed resistance to flumequine, thiamphenicol, florfenicol, sulfamonomethoxine, and trimethoprim/sulfamethoxazole.

## 4. Discussion

The dual-gene identification strategy used in this study (16S rRNA and *gyrB*) aligns with current recommendations for reliable *Aeromonas* species identification, as single-gene approaches based solely on 16S rRNA have limited discriminatory power for closely related species [30,31]. Strain Ag001 was identified as *A. salmonicida* through 16S rRNA (99.9% identity with AB027544.2) and *gyrB* gene (100% identity with KC244775.1) sequencing, and its pathogenicity was further confirmed through artificial infection experiments. In addition, *vapA* gene phylogenetic analysis further supported the classification of Ag001 as *A. salmonicida* subsp. *salmonicida*. MLST has become an important tool for molecular epidemiological surveillance because of its higher discriminatory power compared with single-locus typing methods [32,33]. Using this six-locus typing scheme, Ag001 was assigned to sequence type ST2 in the *Aeromonas* pubMLST database. Previous studies have shown that strains belonging to this clonal lineage are associated with disease outbreaks in *Salmo salar* [34]. Our findings further expand the known host range of this pathogen to include *T. arcticus grubei*, a previously unreported salmonid host species.

Histopathological analysis accurately reflects pathological alterations at the tissue and cellular levels and provides important evidence for disease diagnosis. In the present study, *T. arcticus grubei* infected with *A. salmonicida* exhibited hemorrhagic skin ulceration and furuncle formation, which are characteristic manifestations of furunculosis in salmonids [35,36]. Splenic lesions included loosely arranged splenocytes, focal necrosis, cytoplasmic vacuolization, and nuclear fragmentation and dissolution. These pathological alterations suggest severe splenic injury and immune cell damage during systemic infection. In the liver, marked congestion, disorganization of hepatic cords, inflammatory cell infiltration, and hepatocyte degeneration were observed, indicating substantial hepatic injury associated with bacterial infection and toxin activity. Renal tissue exhibited disorganized renal tubules, epithelial cell degeneration and necrosis, and scattered hemorrhagic foci, suggesting impaired renal structure and function. Collectively, these histopathological findings are consistent with previous reports of systemic *A. salmonicida* infection in salmonids and further support the strong pathogenic potential of Ag001 in *T. arcticus grubei* [37,38,39].

Challenge experiments demonstrated that Ag001 exhibits high pathogenicity toward both *T. arcticus grubei* and *O. mykiss*. Although differences in experimental conditions limit direct quantitative comparisons among independent studies, comparison with the recently reported *A. salmonicida* strain SL0n [37] revealed markedly lower LD_50_ values for Ag001. The LD_50_ of Ag001 in *O. mykiss* was 3.46 × 10^1^ CFU/g, whereas the SL0n strain exhibited an LD_50_ of approximately 1.63 × 10^6^ CFU/fish (1.15 × 10^4^ CFU/g). The limited number of fish per dose group and the use of grouped mortality data in the improved Kärber analysis may affect the precision of the LD_50_ estimates obtained in this study. Moreover, the use of a single tank for each treatment group represents a limitation of the experimental design, as potential tank effects could not be independently evaluated. This apparent difference in virulence may be associated with genomic differences between the two strains. Comparative genomic analysis revealed that Ag001 carries both a complete chromosome-encoded T2SS and a plasmid-encoded T3SS, whereas the SL0n strain lacks the T3SS-associated plasmid. Whether this genomic distinction contributes to the apparent difference in virulence between the two strains remains to be determined through standardized comparative infection experiments and functional analyses. Previous studies have consistently demonstrated the important contribution of the T3SS to the virulence of *A. salmonicida*. Origgi et al. reported that disruption of the T3SS-associated ascV gene resulted in a marked attenuation of virulence and loss of lethality in fish infection experiments [40]. Similarly, Wang et al. demonstrated that loss of the T3SS plasmid in *A. salmonicida* subsp. *masoucida* increased the LD_50_ by more than 10,000-fold [41]. These findings are consistent with the substantially lower LD_50_ observed for Ag001 in the present study. In *Aeromonas* spp., the T2SS is recognized as a classical secretion system responsible for exporting extracellular virulence factors such as *AerA* and *HlyA*, which contribute to host colonization, tissue injury, and cytotoxicity [42,43,44]. In contrast, the T3SS mediates the direct translocation of effector proteins into host cells and has been implicated in immune modulation during infection. For example, AopP has been reported to interfere with NF-κB signaling and inflammatory responses [45,46], whereas AopN suppresses interferon-γ-associated immune activity [47]. Collectively, these observations suggest that the plasmid-encoded T3SS may contribute to the virulence of Ag001, although its expression and functional involvement in pathogenicity were not directly examined in the present study.

Complementing the secretion system analysis, we characterized the repertoire of individual virulence-associated genes in Ag001. PCR analysis identified eight virulence-associated genes (*aerA*, *act*, *zot*, *dna*, *sp*, *lip*, *vapA*, and *hlyA*) in Ag001, suggesting that these genetic factors may be associated with its pathogenic potential, although their expression and functional roles during infection remain to be determined. Among the various virulence factors, *A. salmonicida* carrying *hlyA* and *aerA* tends to be highly pathogenic [23,48,49]. Aer, encoded by the *aerA* gene, is a hemolytic, cytotoxic, and enterotoxic toxin that interacts with specific glycoprotein receptors on host cell membranes, leading to membrane disruption and cellular damage [50]. Consistently, deletion of *aerA* significantly attenuated the virulence of the original strain [51]. HlyA collaborates with AerA to create membrane pores, leading to cell lysis [23]. This cytolytic activity is further amplified by Act, a multifunctional toxin that drives multi-organ damage through its hemolytic, cytotoxic, and enterotoxic activities [52,53]. Beyond these secreted effectors, structural and enzymatic factors play essential supporting roles. The A-layer, encoded by the *VapA* gene, serves as a core virulence factor that facilitates host cell attachment and invasion [54], thereby laying the foundation for subsequent toxin attacks. Concurrently, the extracellular lipase encoded by *lip* enhances infection severity by modifying the host plasma membrane [55]. Collectively, the presence of these virulence-associated genes provides genetic evidence of the pathogenic potential of Ag001 and suggests several candidate factors that may contribute to the tissue lesions observed during infection. Nevertheless, their expression, interactions, and individual contributions to the pathogenic phenotype of Ag001 remain to be experimentally determined.

The localization of the T3SS gene cluster on plasmid A raises the possibility that horizontal gene transfer may have contributed to the acquisition or dissemination of this virulence-associated locus. However, horizontal gene transfer was not directly investigated in the present study, and this interpretation therefore remains a hypothesis that requires further comparative genomic and experimental validation. Ag001 belongs to the ST2 lineage, which has previously been associated with disease outbreaks in Atlantic salmon aquaculture [34], supporting the possibility of dissemination of virulence-associated genotypes in aquaculture environments.

Given the clinical implications of this virulent strain, we evaluated its antimicrobial susceptibility to inform potential treatment strategies. Bacterial drug resistance is a significant factor affecting the susceptibility of hosts to infection [56]. Ag001 showed reduced susceptibility to flumequine, thiamphenicol, florfenicol, sulfamonomethoxine, and trimethoprim/sulfamethoxazole among the eight antibiotics tested, while remaining sensitive to enrofloxacin, neomycin, and doxycycline. Enrofloxacin, with an MIC of 0.5 μg/mL, inhibited Ag001 growth under the test conditions and may represent a potential treatment option for the ongoing *T. arcticus grubei* outbreak. However, limited pharmacokinetic data and the lack of established dosing regimens for this species indicate that further pharmacological and clinical studies are necessary to establish safe and effective treatment strategies.

## 5. Conclusions

This study reports the first isolation and characterization of a virulent *A. salmonicida* subsp. *salmonicida* ST2 strain from diseased *T. arcticus grubei* in China. Combined pathogenicity assays and whole-genome analysis demonstrated that strain Ag001 exhibits high virulence toward both *T. arcticus grubei* and *O. mykiss*. Comparative genomic analysis suggested that the presence of a plasmid-encoded T3SS, together with multiple classical virulence-associated genes, may contribute to its pathogenicity. Antimicrobial susceptibility testing further indicated that Ag001 remains susceptible to enrofloxacin, neomycin, and doxycycline, providing information for disease-control planning. These findings expand the known host range and epidemiological knowledge of the *A. salmonicida* ST2 lineage and provide a scientific basis for disease surveillance and health management in cold-water aquaculture systems.

## Figures and Tables

**Figure 1:**
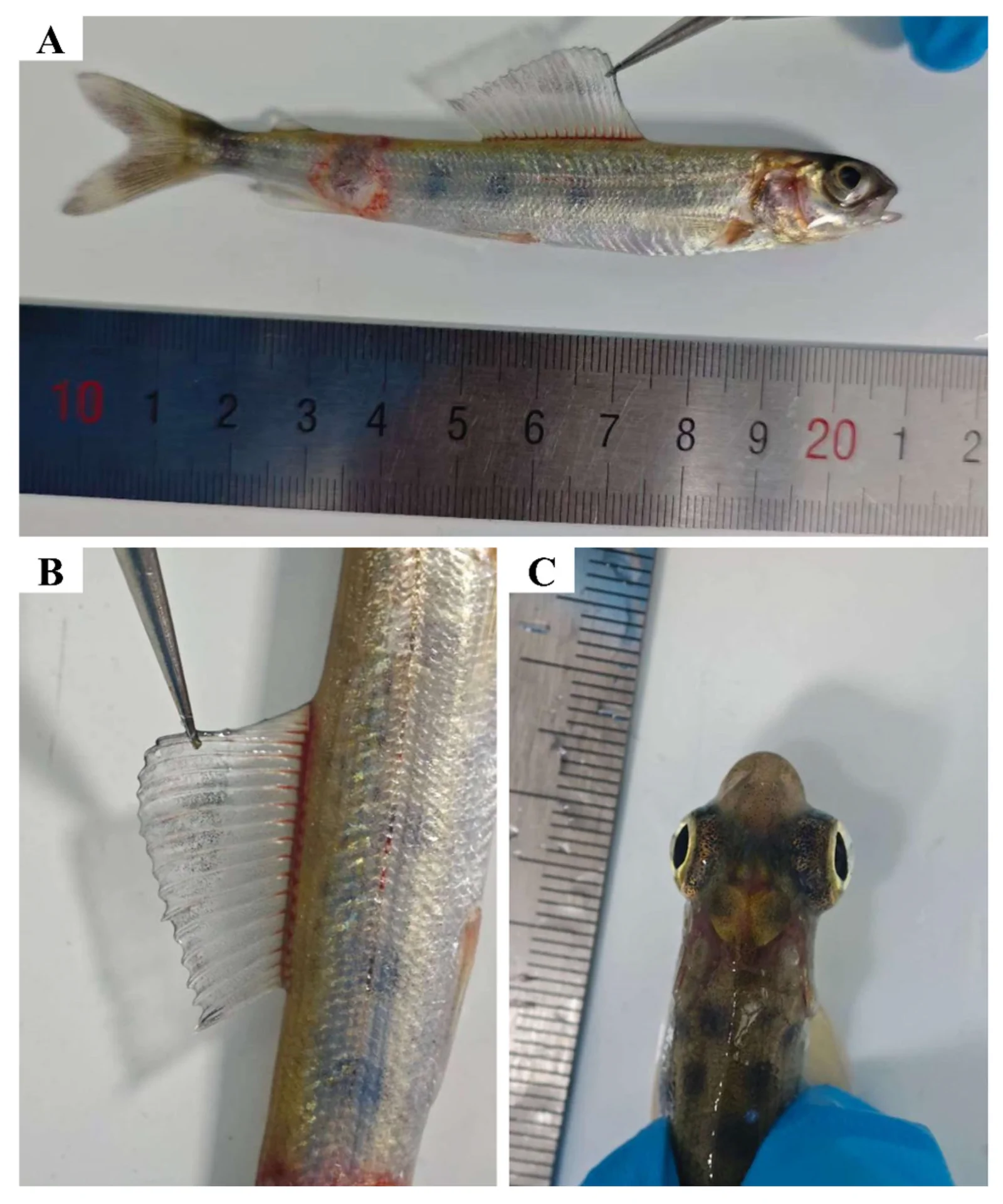
Clinical symptoms of the diseased *T. arcticus grubei*. (**A**) Ulcerative hemorrhagic furuncle with tissue necrosis on the lateral body surface. (**B**) Hyperemia and hemorrhage at the base of the dorsal fin. (**C**) Bilateral exophthalmia accompanied by subcutaneous cranial hyperemia.

**Figure 2:**
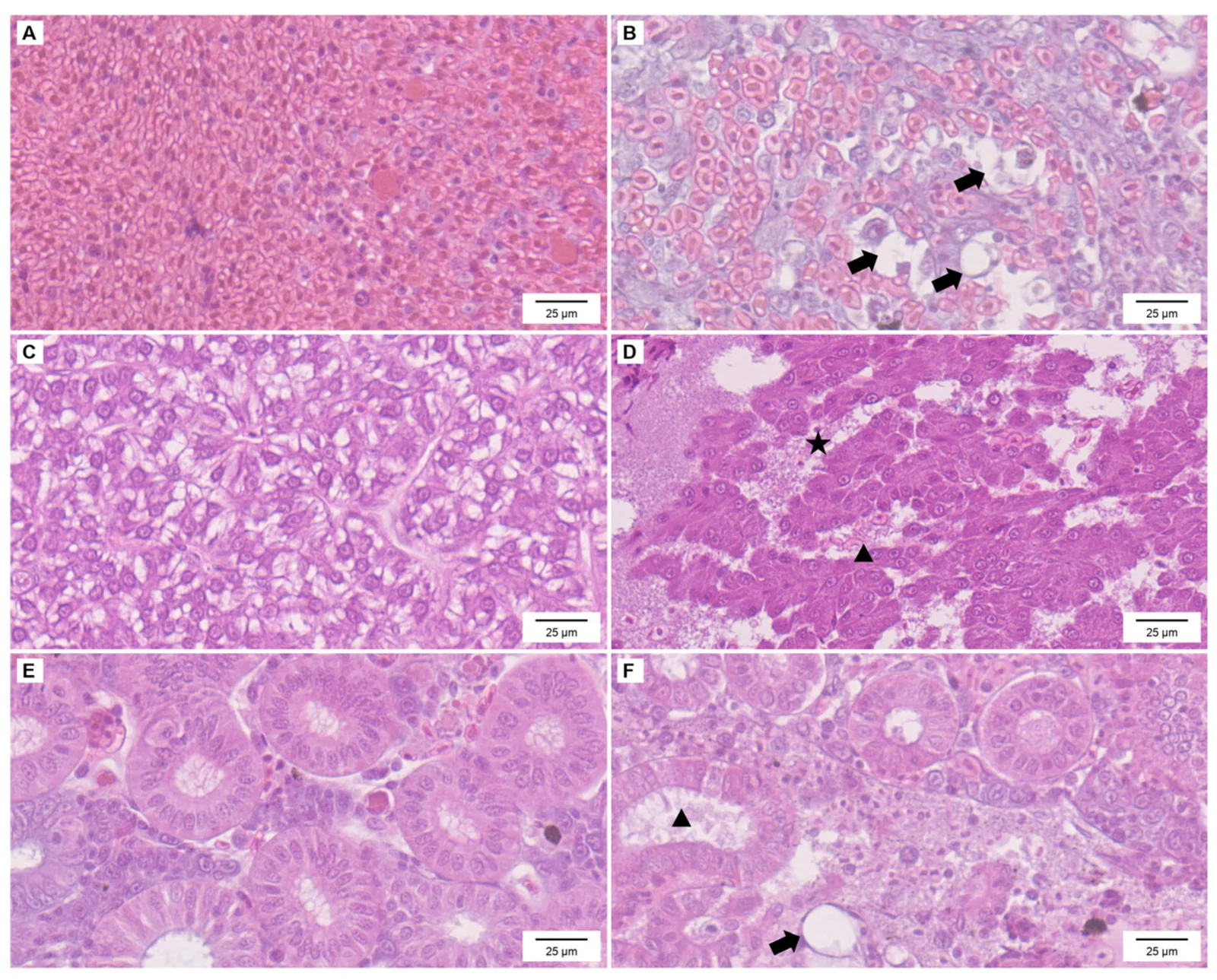
Histological observation of the tissues of healthy and diseased *T. arcticus grubei*. (**A**) Normal spleen tissue; (**B**) spleen tissue from diseased *T. arcticus grubei* showing splenocyte vacuolization (arrow); (**C**) normal liver tissue; (**D**) diseased liver showing focal loss of hepatic parenchymal continuity (triangle) and inflammatory cell infiltration (asterisk); (**E**) normal kidney tissue; (**F**) kidney tissue from diseased *T. arcticus grubei* showing vacuolization (arrow) and loss of normal renal tubular architecture (triangle). Representative sections are shown from healthy and diseased fish (*n* = 3 fish per group). Scale bars = 25 μm.

**Figure 3:**
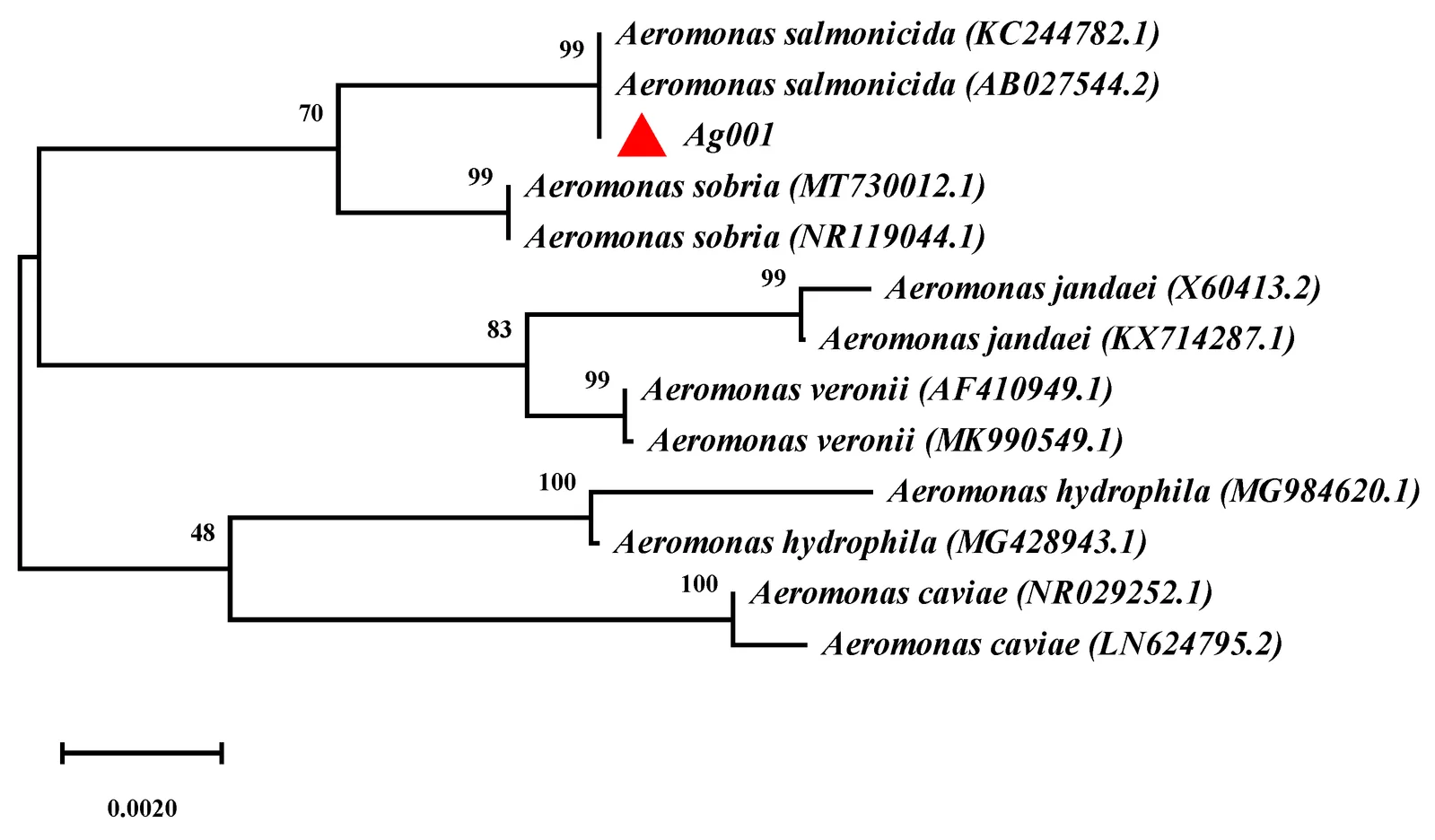
Phylogenetic relationships based on 16S rRNA gene sequences of Ag001.

**Figure 4:**
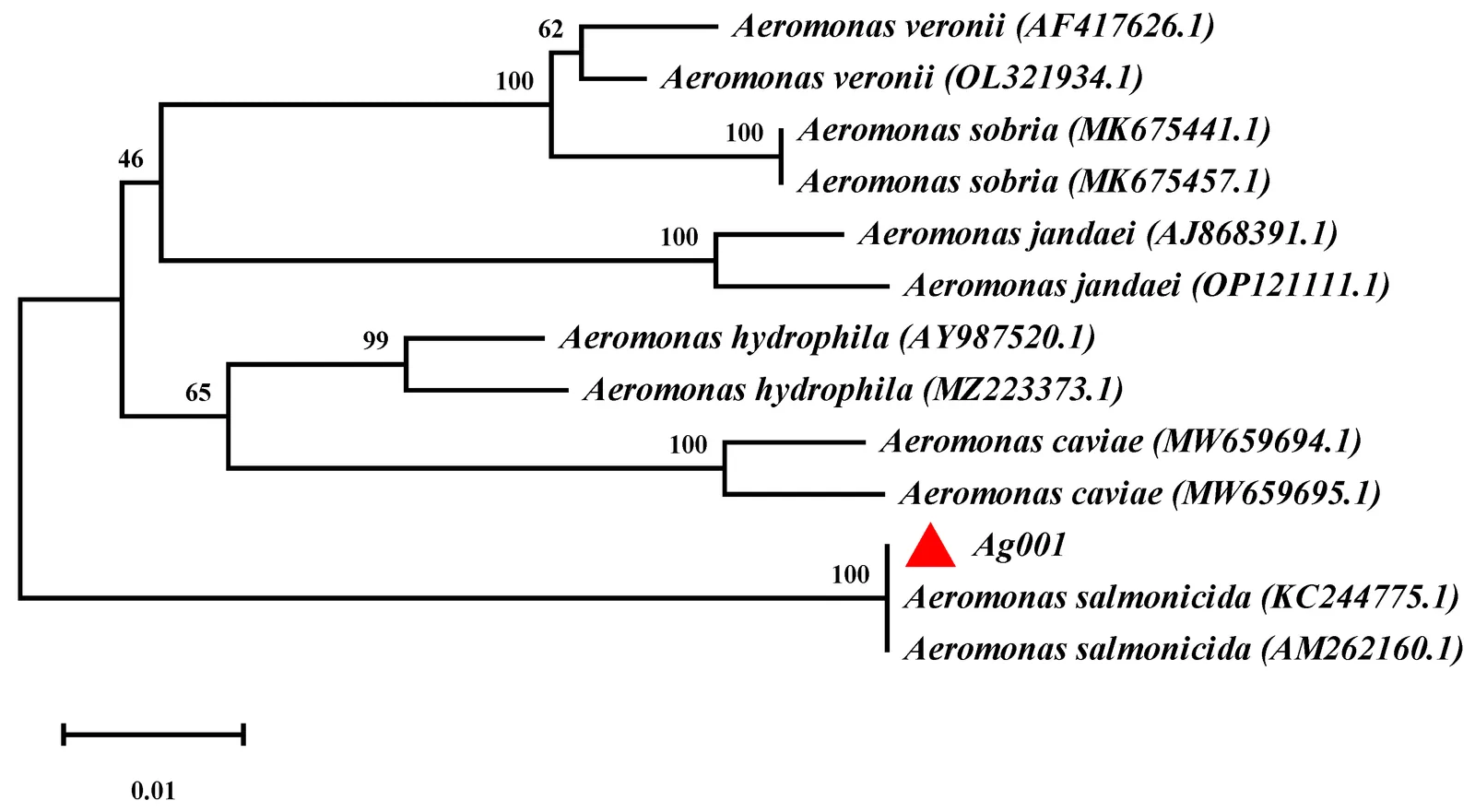
Phylogenetic relationships based on *gyrB* gene sequences of Ag001.

**Figure 5:**
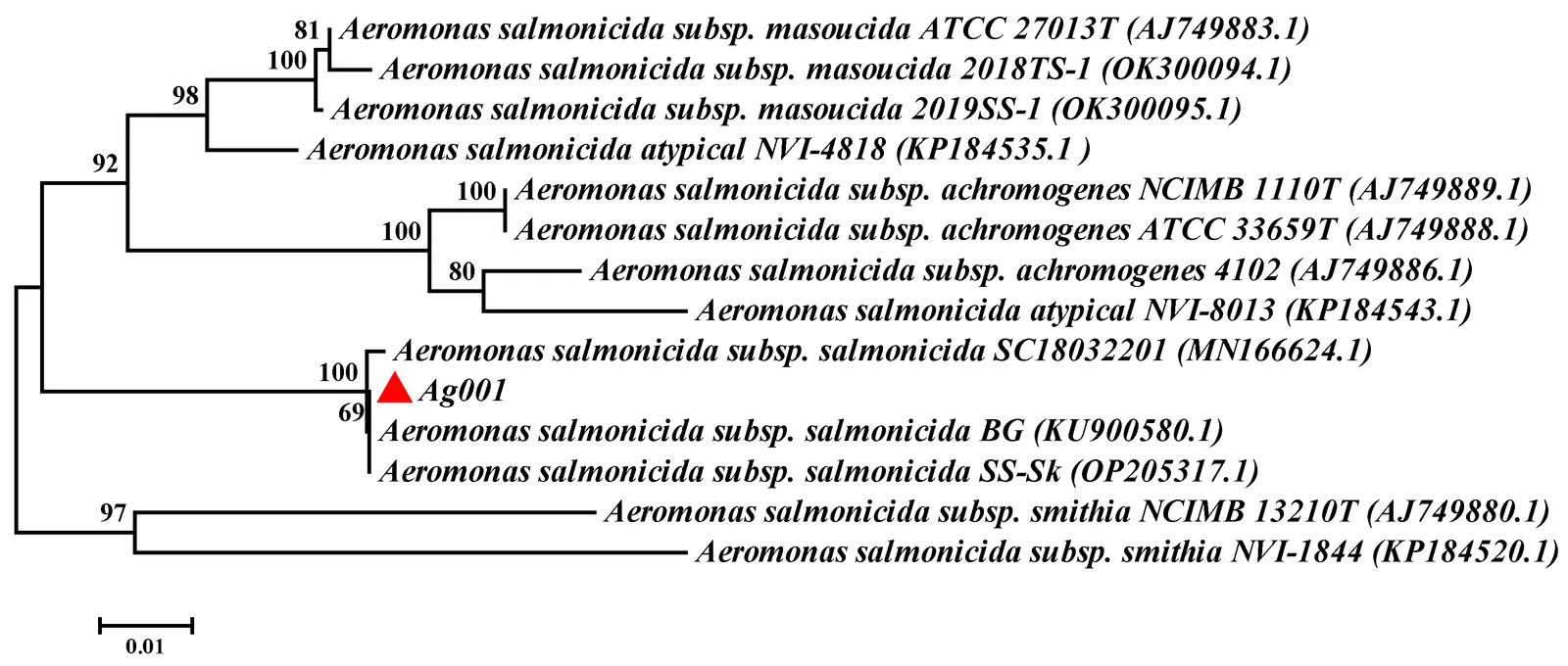
Phylogenetic relationships based on *vapA* gene sequences of Ag001.

**Figure 6:**
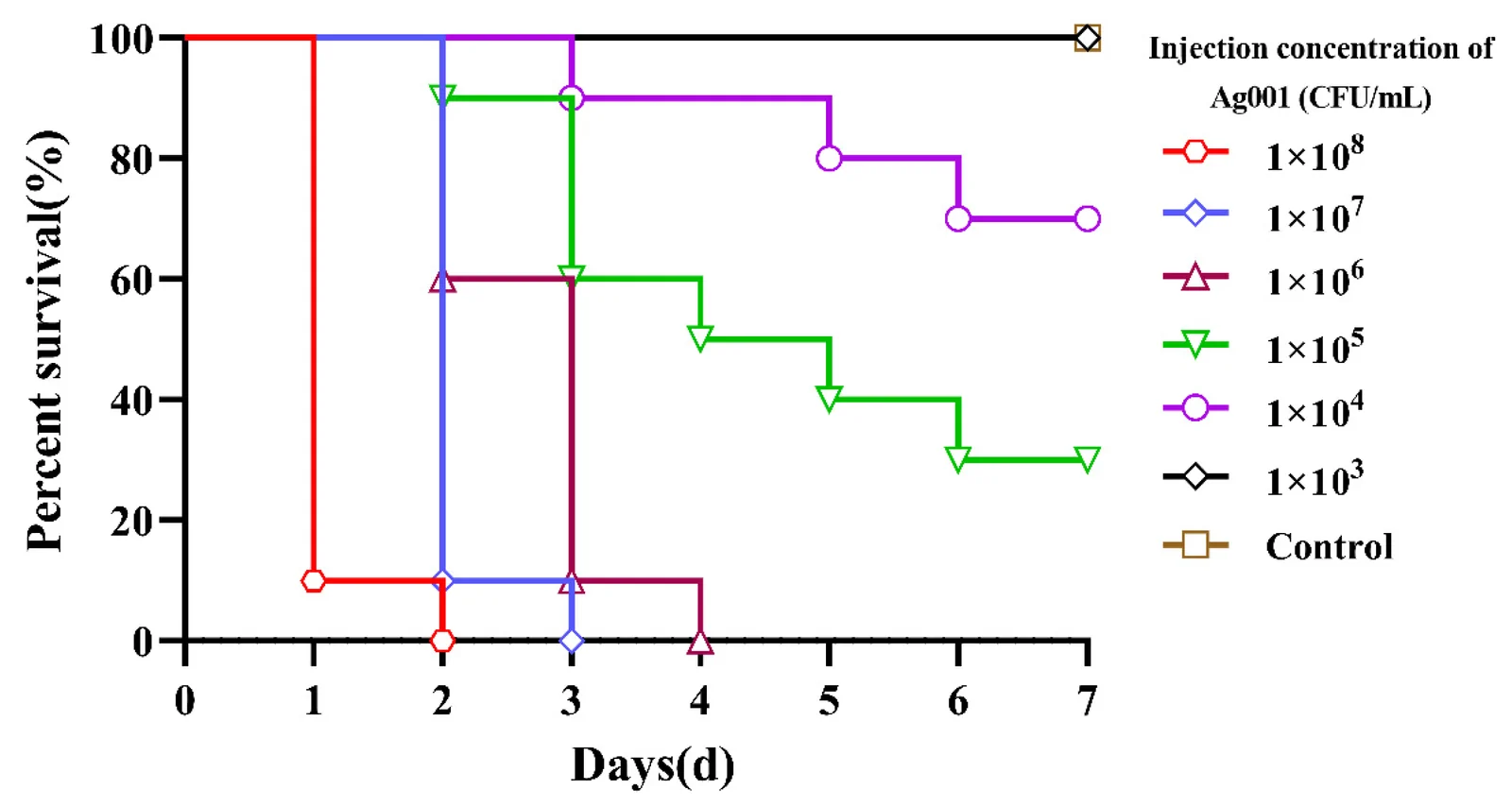
Cumulative mortality of *O. mykiss* challenged with different concentrations of Ag001. Each dose group and the PBS control group contained 10 fish (*n* = 10).

**Figure 7:**
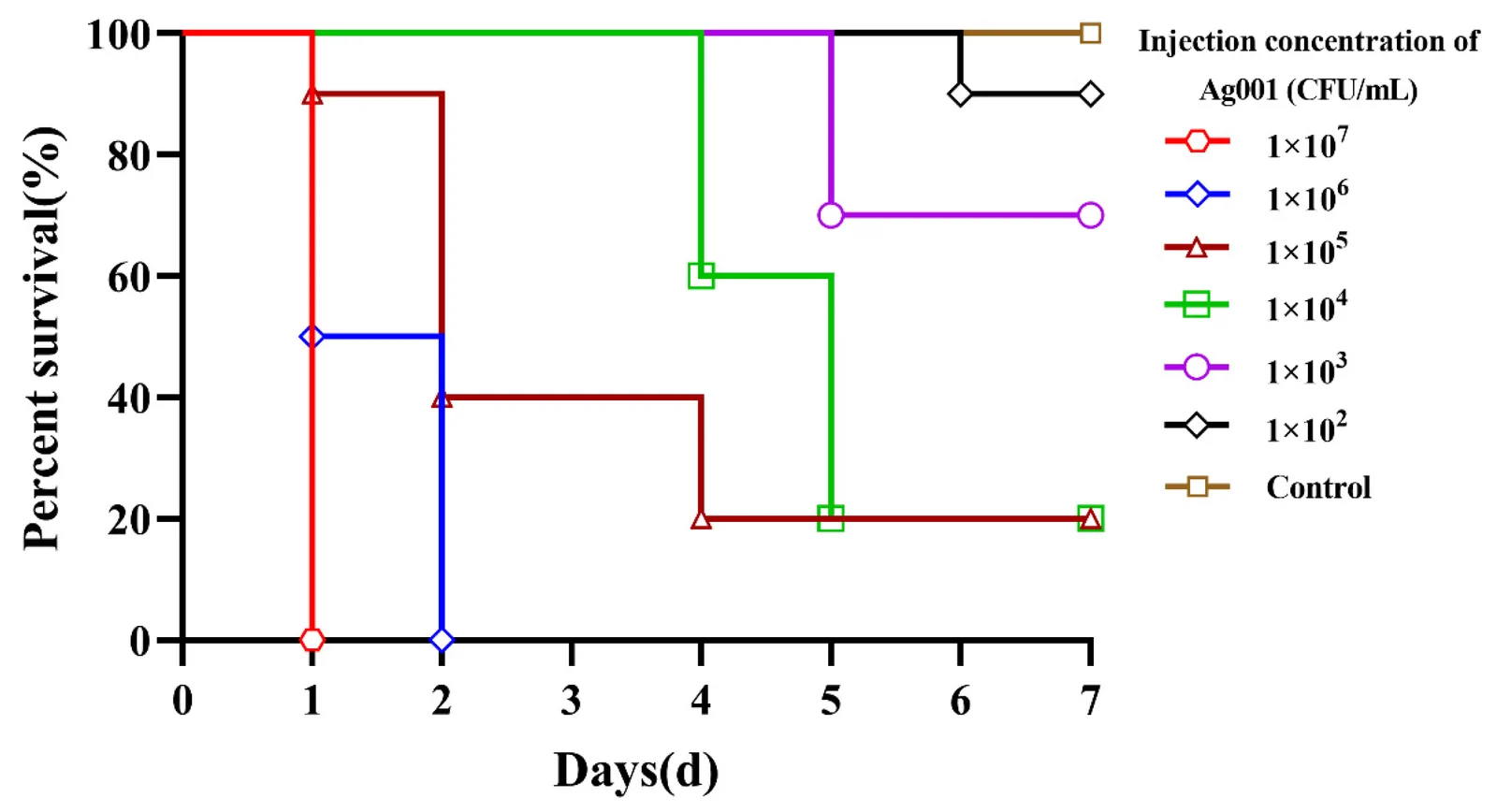
Cumulative mortality of *T. arcticus grubei* challenged with different concentrations of Ag001. Each dose group and the PBS control group contained 10 fish (*n* = 10).

**Figure 8:**
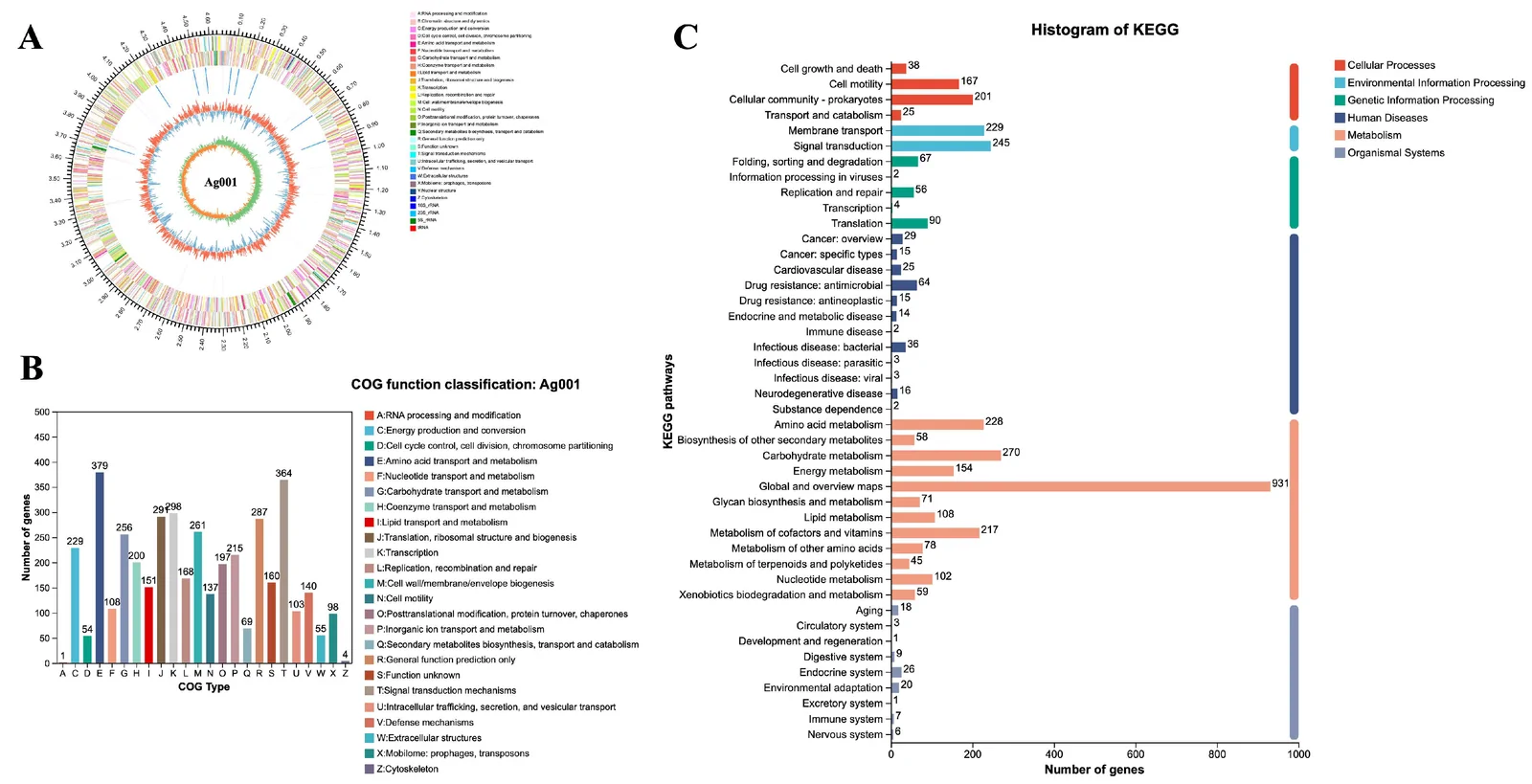
Genome sequencing and annotation of strain Ag001. (**A**) Schematic representation of the strain Ag001 chromosome. From outside to inside: the first circle is the identification of genome size; the second and third circles are protein-coding sequences on the positive and negative strands (different colors indicate the functional classification in COG categories); the fourth circle represents rRNA and tRNA; the fifth and sixth circles mean the GC content and the GC-Skew value; (**B**) COG classification of the genome of strain Ag001; (**C**) elucidation of KEGG pathway annotation.

**Figure 9:**
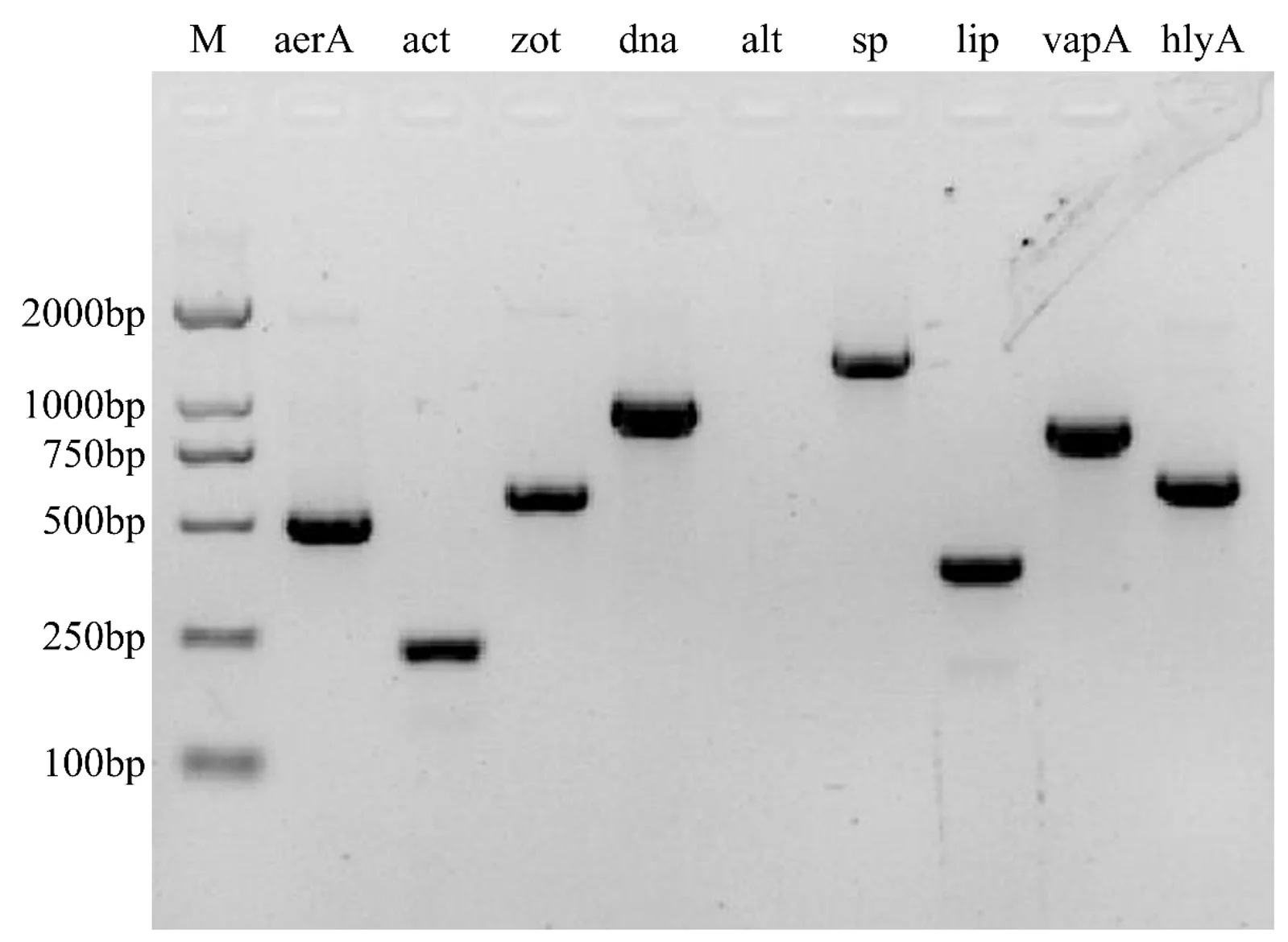
PCR detection of nine virulence-associated genes in strain Ag001.

**Table 1:** Primers for PCR detection of 9 virulence-associated genes.

Gene	PCR Primer Sequence (5′ → 3′)	Product Size (bp)	Reference
*aerA*	F: CCCGCCGATCTGCAACCGGG	489	[21]
	R: CTGGTCTGGATAGACGGGCTCTGCC		
*act*	F: AGAAGGTGACCACCAAGAACA	232	[22]
	R: AACTGACATCGGCCTTGAACTC		
*zot*	F: ACGGTCACAACGGCAGTTACAA	576	[23]
	R: CCACTTCACAGCAGGCACGAA		
*dna*	F: GGCAAGTTTGAATCCGAGCAG	950	[23]
	R: TCGCCGTAGCCGTTGTTC		
*alt*	F: CATGCAGGCAGGCAAGAAGGT	420	[23]
	R: AAGGTCGGCGAGTTCAGCAG		
*sp*	F: CAAGGCTGGCTGACTGCTCAT	1292	[23]
	R: CAAGTGCTGTGGACGCTGGAT		
*lip*	F: ATCTTCTCCGACTGGTTCGG	382	[24]
	R: CCGTGCCAGGACTGGGTCTT		
*vapA*	F: TCGTTACTACCTCCCTCGCA	822	[25]
	R: CACCCAAGACAGCTGTAGCA		
*hlyA*	F: GGCCGGTGGCCCGAAGATACGGG	597	[26]
	R: GGCGGCGCCGGACGAGACGGG		

**Table 2:** The biochemical characteristics of the isolated Ag001.

Characteristics	Ag001	*A. salmonicida* [29]
Gram Stain	−	−
L-Arabinose	+	+
D-Arabinose	−	−
Adonitol	−	−
Glucose (gas)	+	+
Sucrose	+	−
Sorbitol	−	−
D-fructose	+	+
Maltose	+	+
D-Melibiose	−	−
L-Rhamnose	−	−

Note: +, positive; −, negative.

**Table 3:** Multilocus sequence typing analysis of Ag001.

Locus	Identity (%)	Allele Length (bp)	Allele
*gyrB*	100	804	*gyrB*-2
*groL*	100	369	*groL*-2
*recA*	100	1041	*recA*-2
*metG*	100	301	*metG*-2
*gltA*	100	269	*gltA*-2
*ppsA*	100	1130	*ppsA*-2

**Table 4:** Virulence-associated genes predicted using the VFDB.

Gene ID	VF Gene	Product	Accession	Identity (%)
gene0584	*exeM*	general secretion pathway protein M	WP_016349344	92.6
gene2448	*atsD*	Type VI secretion system protein	WP_164927604	71.8
gene0404	*mshO*	MSHA (mannose-sensitive hemagglutinin) pili biogenesis protein MshO	WP_016349166	81.7
gene0673	*tapT*	twitching ATPase	WP_011707389	98.5
gene1541	*flrC*	transcriptional activator	WP_011706626	97.1
gene1524	*flgE*	flagellar hook protein FlgE	WP_016351321	93.5
gene1414	*flpF*	traffic ATPase	WP_016350152	93.6
gene1519	*cheV*	CheV	WP_011706646	96.7
gene1419	*flpJ*	Flp pilus pseudopilin	WP_016350156	91.7
gene0527	*fimF*	fimbrial protein	WP_016349296	83.6

**Table 5:** Drug susceptibility of Ag001 to 8 antimicrobial drugs.

Group	Antibiotic	MIC (μg/mL)	Sensitivity
4-quinolones	Enrofloxacin	0.5	S
	Flumequine	8	R
Chloramphenicols	Thiamphenicol	512	R
	Florfenicol	32	R
Sulfonamides	Sulfamonomethoxine	1024	R
	Trimethoprim/Sulfamethoxazole	64/1216	R
Aminoglycosides	Neomycin	2	S
Tetracyclines	Doxycycline	0.25	S

Note: Sensitivity is designated as S = sensitive and R = resistant.

## Data Availability

The 16S rRNA, *gyrB*, and *vapA* gene sequences of *A.salmonicida* strain Ag001 have been deposited in GenBank under accession numbers PX664495, PX682281, and PZ122746, respectively. The whole-genome sequence of strain Ag001 has been deposited in GenBank under accession number JBVPZQ000000000.1. Other data supporting the findings of this study are available within the article.

## Supplementary Materials

The following supporting information can be downloaded at: https://www.mdpi.com/article/10.3390/biology15191791/s1, Figure S1: Original uncropped PCR gel image corresponding to Figure 9; Table S1: Dose–mortality data used for LD_50_ estimation of strain Ag001.
