# Supplementary material for: Isolation, Pathogenicity, and Genomic Analysis of a Virulent Aeromonas salmonicida ST2 Strain from Thymallus arcticus grubei
Source: Biology (Basel). 2026 Oct 8;15(19):1791. doi: 10.3390/biology15191791 (PMC13664078; doi:10.3390/biology15191791)
Supplement: Supplementary file 1 [file biology-15-01791-s001.zip › biology-4578581-supplementary.pdf]

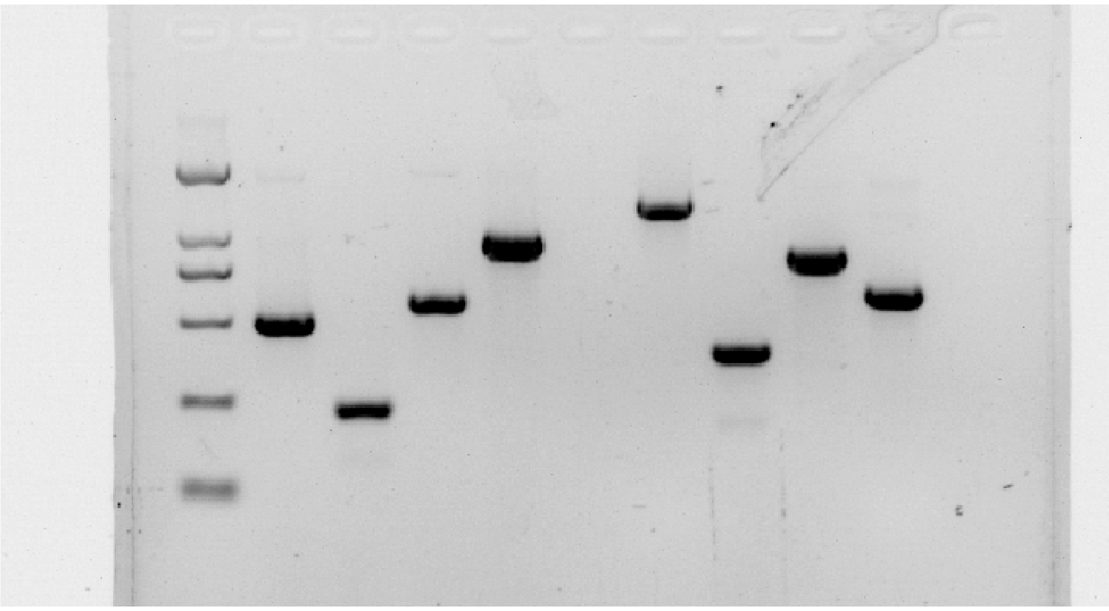

Figure S1. Original uncropped PCR gel image corresponding to Figure 9.

Table S1. Dose–mortality data used for LD50 estimation of strain Ag001.

Species	Bacterial concentration (CFU/mL)	No. challenged	No. dead	Mortality (%)
O. mykiss	1×10^8	10	10	100
	1×10^7	10	10	100
	1×10^6	10	10	100
	1×10^5	10	7	70
	1×10^4	10	3	30
	1×10^3	10	0	0
T. arcticus grubei	1×10^7	10	10	100
	1×10^6	10	10	100
	1×10^5	10	8	80
	1×10^4	10	8	80
	1×10^3	10	3	30
	1×10^2	10	1	10
